# An Evolutionary Cancer Epigenetic Approach Revealed DNA Hypermethylation of Ultra-Conserved Non-Coding Elements in Squamous Cell Carcinoma of Different Mammalian Species

**DOI:** 10.3390/cells9092092

**Published:** 2020-09-13

**Authors:** Luca Morandi, Silvia Sabattini, Andrea Renzi, Antonella Rigillo, Giuliano Bettini, Eva Dervas, Alexandria Schauer, Marco Morandi, Davide B. Gissi, Achille Tarsitano, Stefania Evangelisti, Caterina Tonon

**Affiliations:** 1 Functional MR Unit, Bellaria Hospital, Department of Biomedical and Neuromotor Sciences, University of Bologna, 40139 Bologna, Italy; marco.morandi4@studio.unibo.it (M.M.); stefani.evangelisti4@unibo.it (S.E.); caterina.tonon@unibo.it (C.T.); 2 Department of Veterinary Medical Sciences, University of Bologna, 40064 Ozzano Emilia, Italy; silvia.sabattini@unibo.it (S.S.); andrea.renzi6@unibo.it (A.R.); antonella.rigillo2@unibo.it (A.R.); giuliano.bettini@unibo.it (G.B.); 3 Institute of Veterinary Pathology, Vetsuisse Faculty, University of Zurich, Winterthurerstrasse 190, CH-8057 Zurich, Switzerland; eva.dervas@uzh.ch; 4 Institute of Veterinary Pathology, Vetsuisse Faculty, University of Bern, 8466 Bern, Switzerland; alexandria.schauer@vetsuisse.unibe.ch; 5 Section of Oral Science, Department of Biomedical and Neuromuscular Sciences, University of Bologna, 40125 Bologna, Italy; davide.gissi@unibo.it; 6 Unit of Oral and Maxillofacial Surgery, Azienda Ospedaliero-Universitaria di Bologna, Department of Biomedical and Neuromotor Sciences, University of Bologna, 40138 Bologna, Italy; achille.tarsitano2@unibo.it; 7 IRCCS Istituto delle Scienze Neurologiche di Bologna, Unità di Neuroimmagini Funzionali e Molecolari, Ospedale Bellaria, 40139 Bologna, Italy

**Keywords:** squamous cell carcinoma, ultra-conserved non-coding elements, DNA methylation, bisulfite sequencing, evolutionary epigenetics

## Abstract

Background: Ultra-conserved non-coding elements (UCNEs) are genomic sequences that exhibit > 95% sequence identity between humans, mammals, birds, reptiles, and fish. Recent findings reported their functional role in cancer. The aim of this study was to evaluate the DNA methylation modifications of UNCEs in squamous cell carcinoma (SCC) from different mammal species. Methods: Fifty SCCs from 26 humans, 17 cats, 3 dogs, 1 horse, 1 bovine, 1 badger, and 1 porcupine were investigated. Fourteen feline stomatitis and normal samples from 36 healthy human donors, 7 cats, 5 dogs, 5 horses, 2 bovines and 1 badger were collected as normal controls. Bisulfite next generation sequencing evaluated the DNA methylation level from seven UCNEs (uc.160, uc.283, uc.416, uc.339, uc.270, uc.299, and uc.328). Results: 57/59 CpGs were significantly different according to the Kruskal–Wallis test (*p* < 0.05) comparing normal samples with SCC. A common DNA hypermethylation pattern was observed in SCCs from all the species evaluated in this study, with an increasing trend of hypermethylation starting from normal mucosa, through stomatitis to SCC. Conclusions: Our findings indicate that UCNEs are hypermethylated in human SCC, and this behavior is also conserved among different species of mammals.

## 1. Introduction

Comparative studies on whole vertebrate genomes identified highly conserved non-coding sequences with length >200 bp, called ultra-conserved non-coding elements (UCNEs) [1,2]. In the first seminal work, Bejerano et al. [1] compared genomes of humans, mice and rats, identifying 481 regions with perfect identity. More recently, Dimitreva et al. [2], using slightly-relaxed criteria with a 95% identity and >200 bp in length, identified a list of 4351 orthologues in 18 vertebrate species, mostly located within intergenic regions (2139) and the rest in non-coding parts of genes (1713 in introns, 499 in Untranslated Regions (UTRs)). Relatively-frequent polymorphisms exist in UCNEs, but their derived alleles are frequently found homozygous (less than 6% occurring at frequency >1%) [3]; additionally, 112 single nucleotide polymorphisms (SNPs) are annotated in the Ensembl genome browser as phenotypes associated with muscular dystrophies, amyotrophic lateral sclerosis, eye-related disorders, or cancers. Recent data indicated that the conservation of at least some UCNEs is of high importance in normal phenotype, which is in agreement with knockout studies [4].

Most UCNEs were found in clusters, and more often than expected by chance near coding regions for transcription factors and molecules involved in development. These features have suggested the hypothesis that UCNEs may be candidate regulatory elements with a crucial role in early stages of vertebrate development and differentiation. They harbor important sequence features, such as binding sites of developmental transcription factors to coordinate the expression of essential genes, which is why they were readily conserved over the long course of evolution [5]. Being frequently located at both fragile sites and genomic regions involved in tumors, they were studied as biomarkers in several types of cancers [6,7]. Interestingly, it has been shown that their expression profiles are tissue- and cancer-specific, providing a new tool to successfully distinguish different cancer types and subtypes [8]. Gene expression may be regulated by epigenetic modifications such as DNA methylation at CpG sites, where a cytosine can be methylated (5 mC) on the 5th position on the pyrimidine ring. Since SNPs increase from 1% in the genome to 15% at CpG sites due to deamination of methylated C giving a mutation from C to T, selection pressure has preserved these ancient CpGs within some UCNEs, which have escaped the rapid loss of CpG sites typically seen throughout vertebrate genomes. In general, UCNEs have lower CpG density than other regions [9], while some UCNEs mapped on specific clusters revealed CpG islands with a possible role in gene expression of these loci. In this study, we identified a small set of UCNEs, which contained a CpG island and were already reported to be differentially expressed in various types of cancer, such as uc.160 [10,11], uc.270 [11], uc.283 [12], uc.328, uc.339 [13], uc.299 [14], and uc.416 [15]. We evaluated the DNA methylation level of all these CpG loci in SCC from different mammalian species, comparing it with related normal samples and with stomatitis when available.

## 2. Materials and Methods

### 2.1. Ethics Statement

All clinical investigations regarding human samples were conducted according to the principles of the Declaration of Helsinki. The study was approved by the local ethics committee (study number 520/2018/Sper/AOUBo, protocol number OB-200). All information regarding the human material used in this study was managed using anonymous numerical codes. 

For all non-human samples, as the research did not influence any therapeutic decision, the approval by an ethics committee was not required. All the histological samples were collected for diagnostic purposes in our routine standard care. Owners gave informed consent and consciously agreed to the use of clinical data and stored biological samples for research purposes. For the brushing of normal oral mucosa, samples were collected for research purposes only upon owners’ informed consent. 

### 2.2. Study Population and Brushing Collection

Oral human brushing specimens were collected from 26 consecutive patients treated surgically for oral squamous cell carcinoma (OSCC). All 26 patients (13 females and 13 males (mean age 71 years, 10 smokers)) were diagnosed and treated at the Department of Biomedical and Neuromotor Sciences, University of Bologna, Section of Oral Sciences and the Maxillofacial Surgery Unit, Sant’Orsola Hospital during the period 2018–2019. Index human SCC locations were the following: in 10 patients the tongue and/or the floor of mouth, in 6 patients the right or left cheek, in 9 patients the hard palate or the gingiva and in 1 patient the inferior lip. Fourteen SCCs were diagnosed at early stage (T1-2N0) whereas 12 SCCs were diagnosed at advanced stage (T3-4N+) according to the *p*-TNM classification of tumors (AJCC 8th edition) [16]. Surgical resection of OSCCs was always performed in accordance with standard treatment practice [17]. Brushing specimens from 36 healthy donors (17 females, 19 males) were collected in a cluster of age, sex, and smoking-habits-matched patients presenting at the University Unit only for dental care, during the same period. In this group we avoided collecting samples with any type of lesion in the oral cavity (infective, reactive, or benign). 

Oral brushing sample collection was performed in the population study as previously described [18,19]; in brief, a cytobrush (manufactured by N.H.M.P. Co., Ltd. PRC EC REP: Shanghai International trading corporation, Hamburg, Germany) was used to collect exfoliated cells from oral mucosa. Each cytobrush sample was placed in a 2-mL tube containing 500 µL of DNA/RNA Shield (Zymo Research, Irvine, CA, USA) for nucleic acid preservation. The whole surface of the lesion was gently brushed with rotation and translation movements. In these patients, oral brushing was always performed before incisional biopsy and samples were enrolled in the population study only after histological confirmation of oral SCC. 

For non-human samples, a retrospective–prospective survey was carried out on medical records of the Service of Veterinary Pathology and Veterinary Teaching Hospital at the Department of Veterinary Medical Sciences (University of Bologna, Italy), at the Institute of Veterinary Pathology, Vetsuisse Faculty (University of Bern), and at the Institute of Veterinary Pathology, Vetsuisse Faculty (University of Zurich, Switzerland). 

Representative histological specimens with a diagnosis of SCC (24 cases) from various locations were collected from as many mammalian species as possible. Additionally, histological cases of feline chronic lymphoplasmacellular stomatitis (14 cases) were also included, in order to compare this condition with feline neoplastic oral mucosa, as a greater number of SCCs was available for this species. All the histological samples were formalin-fixed and paraffin-embedded (FFPE), sectioned at 4 μm, and stained with hematoxylin and eosin (HE). 

To obtain controls for as many species as possible with SCC cases, 20 brushing samples of oral mucosa were collected from animals received for clinical visits or autopsies. Only animals without clinical and macroscopic evidence of oral lesions were sampled. The sampling was performed with the same technique as human brushing. Table 1 summarizes clinical data of non-human samples.

Genetic analyses were performed at IRCCS Istituto delle Scienze Neurologiche di Bologna, Department of Biomedical and Neuromotor Sciences, Bellaria Hospital, University of Bologna, Italy.

### 2.3. Ultra-Conserved Non-Coding Element Selection and Primer Designing

In order to identify putative CpG islands present on UCNEs, genomic sequences stored on UCNEbase database [2] and in the Bejerano G et al. collection [1] were employed as query sequences. A set of previously-identified UCNEs that presented epigenetic modifications in tumors were selected for this study: uc.160+ (ID: 27423; downstream gene: *AP3B1*) [10], uc.283 promoter and uc.283 (ID: 6661; downstream gene: *ERCC6*) [12], uc.416 (ID: 16299; within *HOXB5* gene) [15], uc.339 (downstream gene: *KIIA1536*) [13], uc.270 (ID: 35244; within *MAPKAP1* gene) [11], uc.299 (within *PAX2* gene), and uc.328 (within *PAX6* gene) [14].

MethPrimer was applied to identify CpGs and the best primers of choice [20] for targeted sequencing. The list of genomic regions, primer sequences and mapping coordinates interrogated in this study are available in Table 2.

### 2.4. DNA Methylation Analysis

DNA methylation analysis was evaluated as previously described [18,19]. In brief, DNA from exfoliating brush specimens was purified using the MasterPure™ Complete DNA Purification Kit (Lucigen, Middleton, WI, USA, cod. MC85200). DNA from 10 μm sections of FFPE tissues (five for each sample) were purified using the QuickExtract™ FFPE DNA Extraction Kit (Lucigen, Middleton, WI, USA, cod. QEF81050) following the protocol described by Gabusi et al. [21]. Fifty to five hundred ng of DNA was treated with sodium bisulfite using the EZDNA Methylation-Lightning™ Kit (ZymoResearch, Irvine, CA, USA, cod. D5031) according to the manufacturer’s instructions. Quantitative DNA methylation analysis was performed by next-generation sequencing for the following genes: uc.160, uc.283, uc.283 promoter, uc.416, uc.339, uc.270, uc.299, and uc.328. Locus-specific amplicon libraries were generated with tagged primers in two steps: a first PCR amplification for target enrichment, and a second shorter amplification session (eight cycles) to allow the barcoding of the template-specific amplicons obtained from the first amplification step using the Nextera™ Index Kit (Illumina, San Diego, CA, USA) [22,23,24,25,26]. Sequencing was conducted on MiSeq sequencer (Illumina, San Diego, CA, USA), according to the manufacturer’s protocol. Each Next Generation Sequencing (NGS) experiment was designed to allocate at least 1 k reads/region, in order to have a depth of coverage of 1000×.

The methylation ratio of each CpG was calculated in parallel by different tools in a Galaxy Project environment (Europe) [27]: FASTQ files were processed for quality control (>Q 30) and for read lengths (>80 bp) by Filter by Quality and Filter FASTQ reads for quality score and length, respectively. Reads were mapped by BWAmeth, generating a bam file which was processed by MethylDackel using hg38 for human samples and Felis_catus_9.0 for feline samples as a reference. This tool created an excel file assigning at each CpG position the exact methylation level; additionally, we adopted in parallel the EPIC-TABSAT web-tool to confirm our data [28] for human samples, but it worked also for other species as the regions investigated were ultraconserved compared to hg38. Once having catalogued methylation levels for all CpGs in an excel file, the evaluation of differential DNA methylation with group comparison was performed by methylation plotter [29], which provides descriptive statistics and basic non-parametric variance analysis (Kruskal–Wallis tests). For each sample and group, a data table summarizing the mean, standard deviation, minimum and maximum was produced (see Supplementary Tables S1–S3). ClustVis [30] was used to create the HeatMap. Receiver operating characteristic (ROC) analysis was performed using the web tool EasyROC (http://www.biosoft.hacettepe.edu.tr/easyROC/). Multiclass linear discriminant analysis was calculated using IBM SPSS Statistics 21 (IBM). The R packages pcaMethods (https://bioconductor.org/packages/release/bioc/html/pcaMethods.html) and OmicCircos (https://bioconductor.org/packages/release/bioc/html/OmicCircos.html), both included in the software project Bioconductor, were used for principal component analysis (PCA) and circular plots, respectively. The original contributions presented in the study are publicly available. This data can be found at GEO (gene expression omnibus) repository (accession: GSE157436).

## 3. Results

### 3.1. Methylation Plotter Analysis

**Figure 1:**
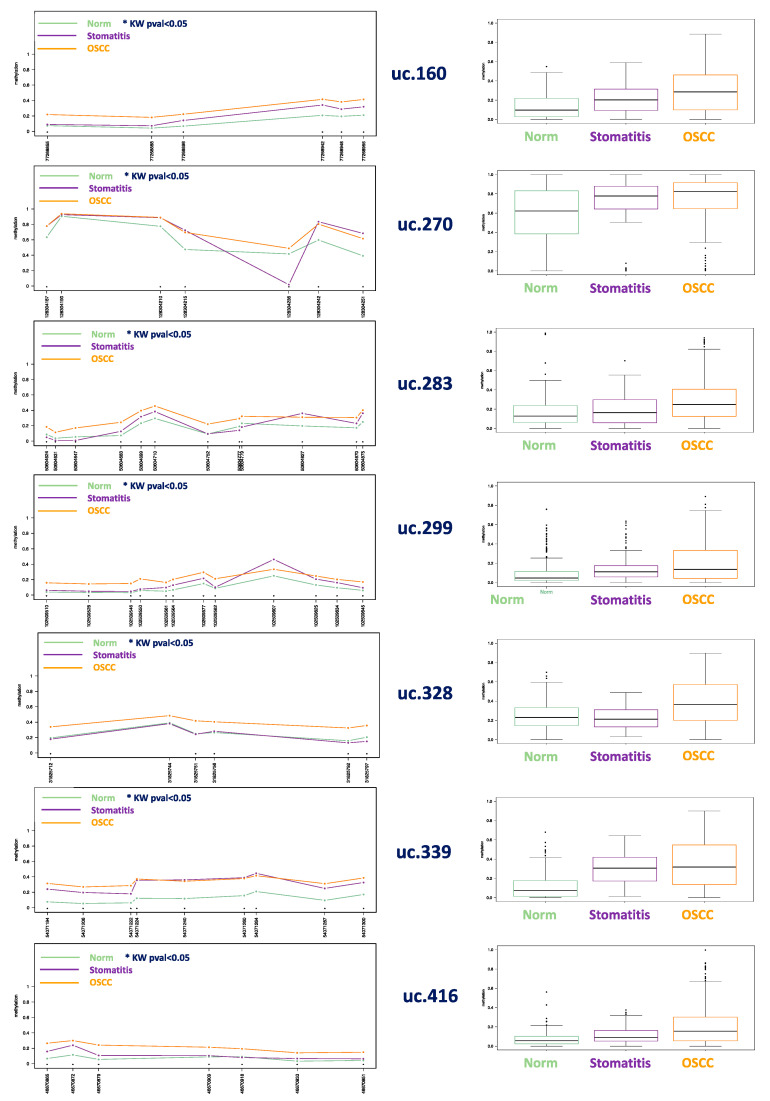
**(Left column**): methylation plots for each of the seven UCNEs comparing all CpG positions (59 in total) in 50 SCCs with 14 feline stomatitis and 62 normal cases from all mammalian species evaluated in this study; the profile plot summarizes the methylation panorama in percentage (values ranging from 0 to 1) comparing the three sample groups, labelling with an asterisk(*) those CpGs that show statistical differences according to the nonparametric Kruskal–Wallis test; uc.160, uc.270, uc.283, uc.299, uc.339, and uc.416 displayed *p*-values < 0.01 in all positions marked with *; uc.328 showed *p* = 0.014 for CpG 31825712, *p* = 0.019 for CpG 31825756, *p* < 0.01 for CpG 31825792, CpG 31825751, and CpG 31825797 (see Supplementary Table S1 for details). (**Right column**): boxplot for each group of samples considering all the CpGs evaluated.

Methylation levels of uc.160, uc.283, uc.416, uc.339, uc.270, uc.299, and uc.328 were assessed in 50 SCCs (from 26 humans, 17 cats, 3 dogs, 1 horse, 1 bovine, 1 badger, and 1 porcupine). Moreover, 14 feline stomatitis and normal tissue from 42 healthy human donors, 7 cats, 5 dogs, 5 horses, 2 bovines, and 1 badger were collected as normal controls. Comparing SCCs from all the species together with normal samples and feline stomatitis, we found a significant hypermethylation pattern of SCCs in all the investigated UCNEs, involving most of the CpGs, as shown in Figure 1. Table S1 (Supplementary Files) summarized all methylation data including Kruskal–Wallis test (*p*-values not adjusted for multiple comparisons are shown). 

We found an increasing trend of methylation from normal tissue, showing the lowest levels, through feline stomatitis, exhibiting an intermediate level, and finally to hypermethylated SCCs, especially for ac.160, uc.299, uc.328, uc.339, and uc.416. In particular, in uc.160 CpG 77268955 the mean level in normal samples was detected as 0.21, 0.32 in feline stomatitis, and 0.41 in SCCs; for uc.299 CpG 102509546 mean level in normal samples was 0.03, 0.05 in feline stomatitis, and 0.15 in SCCs; uc.328 CpG 31825756 showed in normal samples 0.26, 0.28 in feline stomatitis, and 0.40 in SCCs; uc.339 CpG 54071206 revealed in normal samples 0.05, 0.19 in feline stomatitis, and 0.26 in SCCs; finally, uc.416 CpG 46670879, reported 0.05 for normal, 0.10 for stomatitis, and 0.21 for SCCs. All data regarding each CpG investigated are described in Supplementary Table S1.

**Figure 2:**
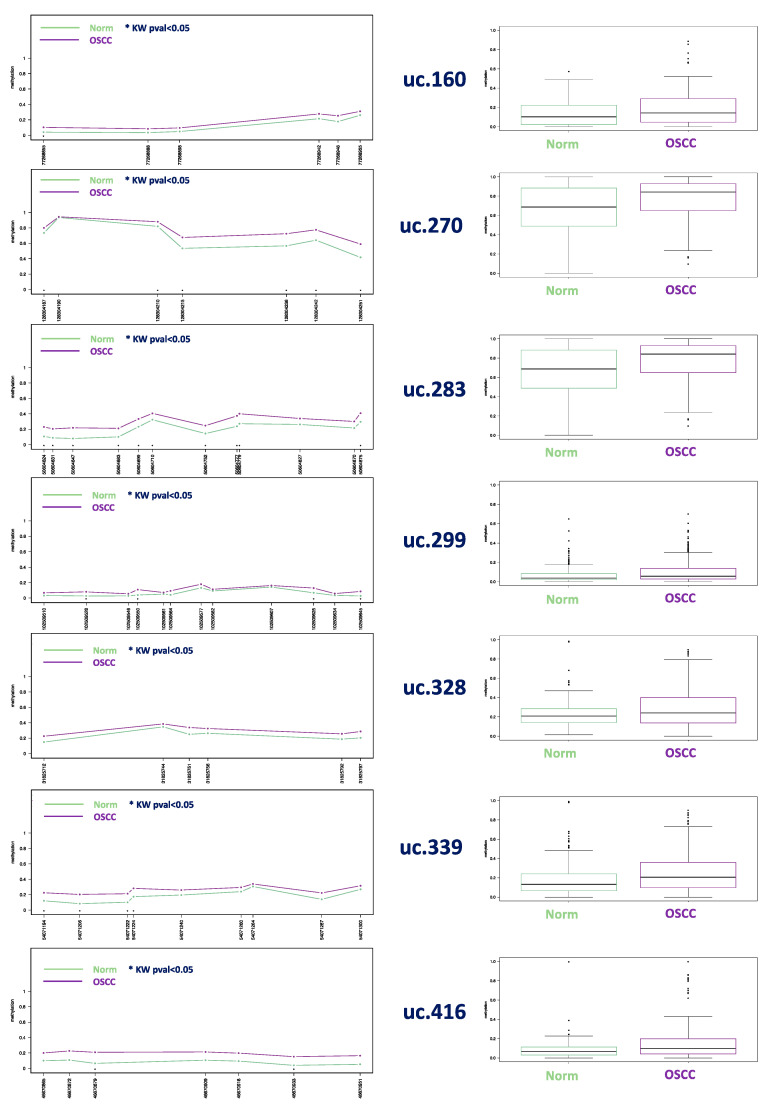
**(Left column**): methylation plot comparing 42 normal donors with 26 SCCs from human samples: statistically significant Kruskal–Wallis test CpG are the following positions: uc.160: CpG 77268855 *p* < 0.01; uc.270: CpG 128304187 *p* = 0.029, CpG 128304210 *p* = 0.016, CpG 128304251 *p* = 0.012, CpG 128304215, 128304236, and 128304242 *p* < 0.01; uc.283: all CpGs * with *p* < 0.01 except for CpG 50604631 (*p* = 0.016), 50604699 (*p* = 0.026), and 50604710 (*p* = 0.042); uc.299: CpG 102509528 and 102509625 showed *p* < 0.01, while *p* = 0.044 was found for CpG 102509550 and *p* = 0.024 for 102509645; uc.339: all CpG with * showed *p* < 0.01 except for CpG 54071224 (*p* = 0.017); uc.416: CpG 46670879 *p* < 0.01, CpG 46670933 *p* = 0.026. (**Right column**): boxplot for each group of samples considering all the CpGs evaluated (see Supplementary Table S2 for details).

The same pattern was observed by considering the single species individually. In brief, comparing human SCCs with normal human samples, hypermethylation of uc.270, uc.283, and uc.339 gave the best discriminative power, with most of the CpGs being statistically significant (Figure 2). As an example, uc.270 CpG 128304236 showed 0.57 as a mean level in normal samples, and 0.72 in SCCs; uc.283 CpG 50604875 showed 0.29 in normal samples and 0.40 in SCCs; and uc.339 CpG 54071222 showed 0.10 in normal samples and 0.21 in SCCs. Evaluating variations within individuals in all normal human samples, only three out of 36 cases showed a distinct pattern of methylation (See the HeatMap in Supplementary Figure S1).

**Figure 3:**
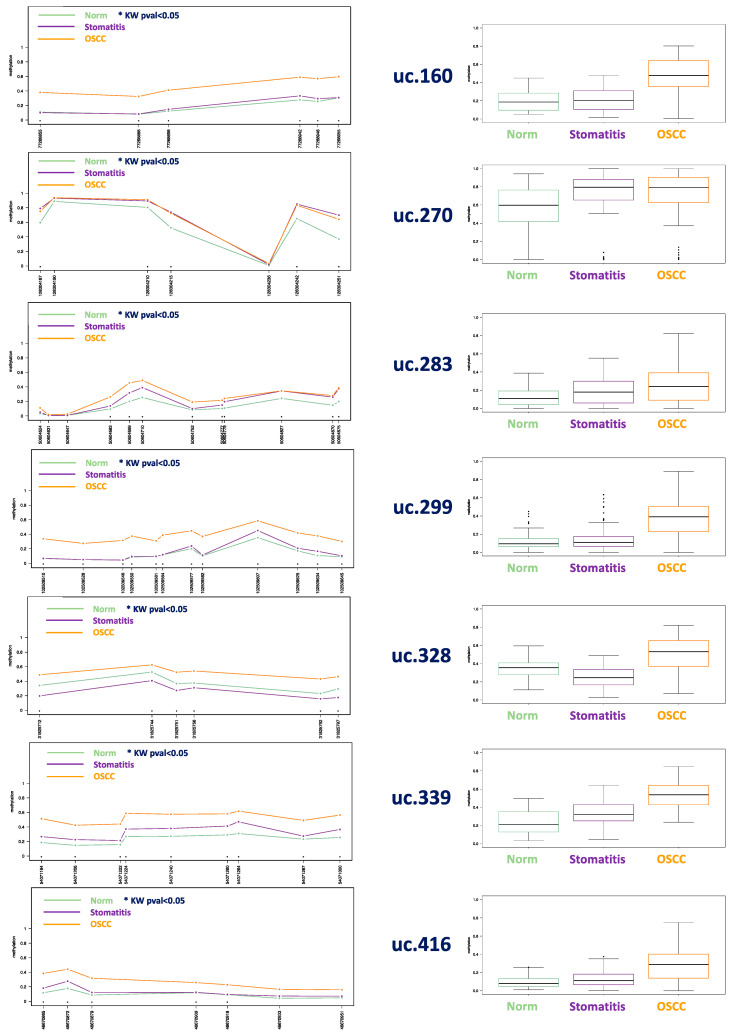
Methylation plot (**left**) and boxplot (**right**) comparing 17 feline SCCs with 14 feline stomatitis, and 7 normal oral epithelium in healthy cats. *p*-values < 0.01 was detected for all CpGs with * mapped in uc.160, uc.270, uc.328, uc.339, uc.416, and uc.283 except for CpG 50604683 (*p* = 0.041), CpG 50604752 (*p* = 0.017), CpG 50604777 (*p* = 0.033), and CpG 50604827 (*p* = 0.049) (uncorrected *p*-values, see Supplementary Table S3 for details).

In cats, all the investigated UCNEs were significantly different between SCCs and non-neoplastic samples (Figure 3). All methylation data are reported in Table S3 (Supplementary Files).

In dogs uc.283, uc.328, and uc.416 showed the best discriminatory potential as shown in Figure S2 (Supplementary File). In this species, uc.339 displayed the lowest level (close to 0) of methylation both for normal samples and SCCs, a condition not observed in the other species. Comparing the equine SCCs with five normal equine samples, uc.160, uc.328, and uc.416 showed the most prominent epigenetic alterations (Figure S3). We also evaluated one bovine SCC against two normal bovine samples detecting hypermethylation in uc.160, uc.270, uc.283, uc.299, and uc.339; the badger SCC, when compared to its relative normal sample showed hypermethylation in uc.160, uc.283, and uc.299; the porcupine SCC, compared to all normal samples of different species, exhibited aberrant methylation in uc.339, uc.270, uc.299, and uc.328.

We also compared DNA methylation levels in SCCs among different species as shown in Figure S4. We found a different methylation level in all the UCNEs investigated. The same different pattern was also detected when comparing normal samples among different species (Figure S5).

### 3.2. Principal Component Analysis (PCA)

**Figure 4:**
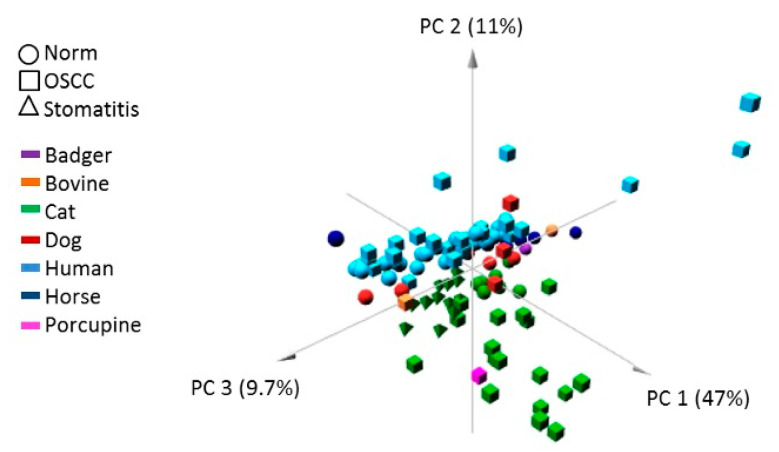
PCA results for the three different types of tissue. Only for display purposes, different species are highlighted with different colors. Unit variance scaling was applied to rows and Singular value decomposition (SVD) with imputation was used to calculate principal components. *X*, *Y,* and *Z* axes show principal components 1, 2, and 3 that explain 47%, 11%, and 9.7% of the total variance, respectively.

A plot showing the first three principal components for PCA with tissue type as grouping factor is shown in Figure 4. SCC elements (square) were quite spread, while normal samples (circle) tended to be more clustered in a well-defined and restricted area, similarly to the stomatitis samples (triangle).

**Figure 5:**
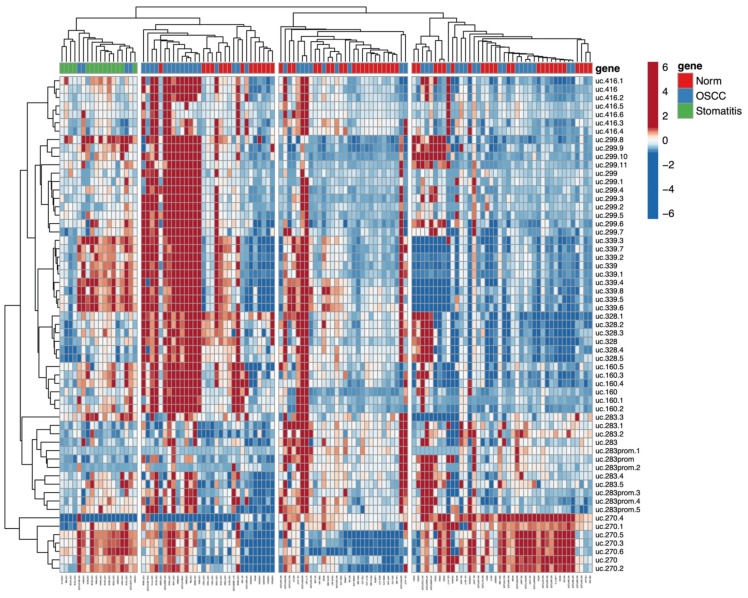
HeatMap evaluating DNA methylation levels of all CpGs and all samples investigated. Values in the matrix are color-coded, and rows (CpGs) and columns (specimens) are clustered using correlation distance and average linkage. Four clusters are marked: starting from the left, cluster 1 comprised all the feline stomatitis and 4 feline SCCs; cluster 2 comprised 17 SCCs (14 cats, 1 horse, 1 porcupine, 1 badger) and 14 normal samples (7 cats, 1 human, 4 horses, 1 bovine, 1 badger); cluster 3 included 10 human SCCs and 20 normal human oral mucosa samples; finally, cluster 4 included 18 SCCs (15 humans, 3 dogs), and 24 normal samples (16 humans, 5 dogs, 1 horse, 1 bovine).

A HeatMap showing a hierarchical clustering related to all the quantitative data and their relationship among all CpGs and all cases was created and shown in Figure 5. Feline stomatitis tend to group together in the same cluster (left side); a second cluster was created with animal SCCs located on the left side and normal animal samples on the right; a third cluster contains only human SCCs (left and right) and normal human oral mucosa samples (middle part); and a 4th cluster includes 18 SCCs (15 humans, 3 dogs), and 24 normal samples (16 humans, 5 dogs, 1 horse, 1 bovine). Clinical features (sex, age, and smoking habits) of the human study population did not influence the clusterization, in particular, for clusters 3 and 4 where human SCCs and normal samples were located. 

**Figure 6:**
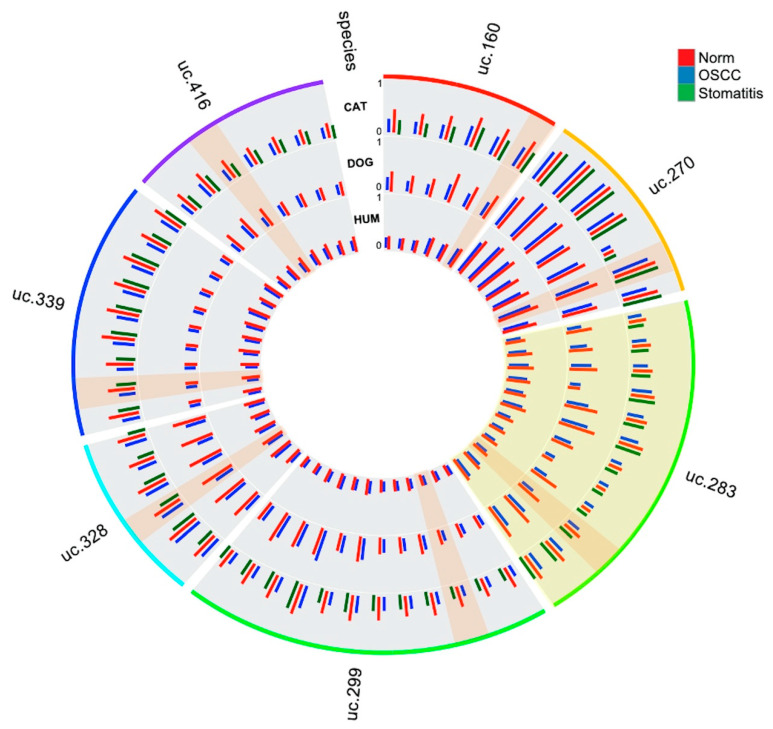
Circular visualization of methylation levels for each of the seven UCNEs in the different tissues for cat, dog, and human samples. UCNE 283 is highlighted as it showed the best discriminative power between SCCs and normal tissues for cats, dogs, and humans. For each UCNE, the best CpG to discriminate SCCs from control samples is also highlighted.

The following circle plot (Figure 6) represents graphically how the methylation level increases from normal donors, through stomatitis to SCC depending on the position of each CpG within the UCNE in humans, cats, and dogs.

### 3.3. Receiver-Operating Characteristic (ROC) Analysis

For each UCNE, the best CpGs to discriminate SCCs from control samples were identified using the EasyROC web tool (Table 3):

We identified uc.416, uc.339, and uc.283 as the best biomarkers with good discriminatory potential area under the curve (AUC): 0.843–0.826. The best CpGs from all UCNEs were used to set up an algorithm of choice based on multiclass discriminant analysis (LDA) that weighted the contribution of each biomarker. The following formula was developed:

Score = 3.586 + (uc.270 ∗ 3.201) + (uc.160 ∗ 0.485) + (uc.328 ∗ 1.618) + (uc.299 ∗ 1.37) + (uc.339 ∗ 0.439) +(uc.416 ∗ 1.892) + (uc.283 ∗ 1.365)(1)

**Figure 7:**
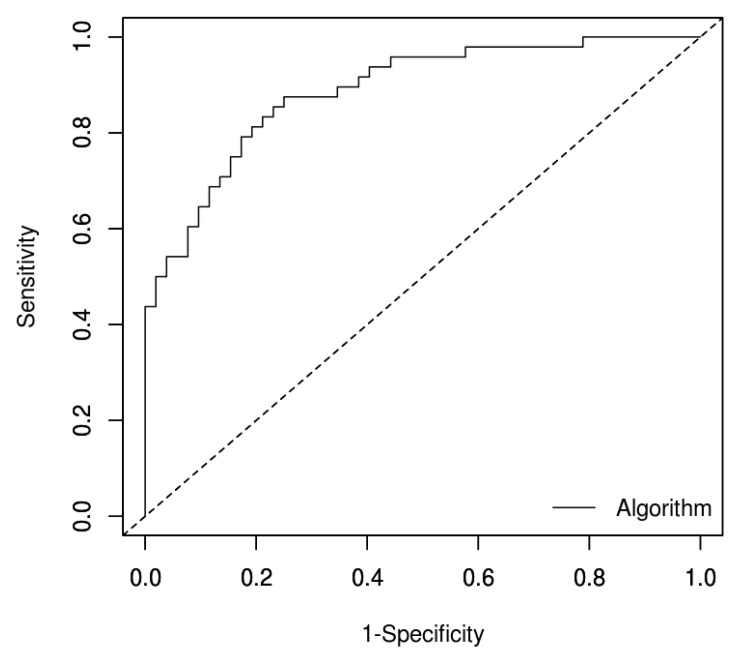
Receiver operating characteristic (ROC) curve analysis based on the algorithm described above: area under the curve (AUC) was calculated as 0.887.

Data coming from these calculations were used to evaluate sensitivity (0.875), specificity (0.750) and the AUC (0.887, Figure 7), with a threshold of −0.232. We were able to correctly identify 20 human and 22 animal SCCs and 13 human and 26 animal normal cases, while we reported 4 human and 8 animal false positive results (of which 7 were stomatitis), and 5 human and 1 animal false negative cases.

## 4. Discussion

In this study, the DNA methylation level of seven CpG-rich UCNEs were investigated in SCC from different mammalian species, including human, cat, dog, horse, bovine, porcupine, and badger. We compared those data with normal samples from the same species and feline stomatitis. Multilevel comparisons highlighted the presence of epigenetic alterations in several CpGs, reaching statistically-significant values using the Kruskal–Wallis test by the methylation plotter tool. These results were obtained by evaluating SCCs altogether vs. all the normal samples and feline stomatitis (Figure 1), but also considering a species–specific comparison between SCCs and normal tissue (Figure 2, Figure 3, Figures S1 and S2). In this regard, we think that the latter is the most reliable method to detect a clear epigenetic aberration, as we identified a variable range of methylation levels among different species both for SCCs and normal samples. These fluctuations are shown in Figure S3 for SCCs and in Figure S4 for normal cases, highlighting the aberrant pattern of DNA hypermethylation in all the investigated UCNEs. This pattern was also confirmed evaluating the PCA (Figure 4) and the HeatMap (Figure 5), showing a first cluster (cluster 1) comprising all the feline stomatitis that were close to each other and four feline SCCs; a second cluster (cluster 2) characterized by feline and non-human SCCs located on the left side and highly hypermethylated in almost all the UCNEs, and normal feline and other non-human normal samples on the right; cluster 3, specific for human SCCs with two groups, one on the left and one on the right side, while normal human oral mucosa samples were located in the middle part; finally, a more heterogeneous cluster (cluster 4) including 18 SCCs (15 humans, 3 dogs), and 24 normal samples (16 humans, 5 dogs, 1 horse, 1 bovine) characterized by hypermethylation of uc.270. Evaluating the relationship between clinical parameters (sex, age, and smoking habits) and hierarchical clustering among groups, we found no correlation. 

Analyzing data from single species, in human SCCs uc.270, uc.283, and uc.339 were identified as the best discriminative biomarkers, with most of the CpGs being statistically significant (6/7, 10/11, and 4/9, respectively). In cats, we were able to retrieve data from a supplementary group made of stomatitis to be compared with normal tissue and SCC, since these inflammatory lesions were recently reported to be altered from an epigenetic point of view [31]. This may be due to an epigenetically-regulated expression of proinflammatory cytokines and other inflammatory-related genes. Moreover, chronic inflammation triggered by various factors can induce aberrant methylation, which in some cases has a preneoplastic effect in epithelial cells [32,33]. In our cohort, we reported a trend of increased methylation starting from normal tissue, through stomatitis to SCC, where the latter exhibited the highest level of methylation. Interestingly, we found the same trend in all the seven UCNEs investigated, all showing most of the CpGs to be statistically significant. In the dog, uc.283, uc.328, and uc.416 revealed the best discriminatory potential; in contrast to other species, uc.339 were completely unmethylated both for normal tissue and for SCC. Unfortunately, we were able to retrieve from our archives only one equine SCC to be compared with five normal samples from the same species, showing uc.160, uc.328, and uc.416 as the most relevant epigenetic biomarkers. Although only one bovine SCC was compared with two normal samples from the same species, we found hypermethylation in uc.160, uc.270, uc.283, uc.299, and uc.339. One case of badger SCC vs. one case of normal tissue showed hypermethylation in uc.160, uc.283, and uc.299. Finally, one case of porcupine SCC could not be compared with normal tissue of the same species, however, comparing it with all the normal samples available from different species, it exhibited an aberrant methylation in uc.339, uc.270, uc.299, and uc.328. 

Taken together, these data indicated a clear hypermethylation status of UCNEs in all the investigated species, with uc.416, uc.339, and uc.283 showing the best AUC values. We also estimated the AUC range of any CpG within each UCNE, since the location of DNA methylation has been reported to have a crucial role in regulating gene expression [18,34]. The best to worse gap varied a little from 0.058 to 0.084 in uc.160, uc.299, uc.339, and uc.328, while we reported higher variations in uc.270, uc.283, and uc.416, ranging from 0.225 to 0.164. Selecting the best CpGs from each UCNE, we were able to calculate an algorithm of choice to better discriminate SCCs from normal samples using a linear discriminant analysis (LDA) approach. The use of a single CpG may be inadequate for this purpose, since the best AUC was detected from uc.416 (AUC: 0.843, coordinate 46670879). The new algorithm slightly increased the performance, since we were able to reach a sensitivity of 0.875, a specificity of 0.750, and an AUC of 0.887, with a threshold of −0.232 for the score. By calculating a score using the developed algorithm, we were able to assign a correct diagnosis in 20 human and 22 animal SCCs, as well as in 13 and 26 normal cases from humans and animals, respectively. However, we reported four human and eight animal false positive results (of which seven were stomatitis) affecting specificity, and five human and one animal false negative cases. Further tests in an independent larger cohort of cases will be necessary to provide strong evidence that these biomarkers can accurately identify SCC. If confirmed, this completely new approach involving the same target regions being present in human and several different mammalian species, could be applied to human and veterinary diagnostics with the same protocol.

One limit of this study was related to the low number of cases for some species which were difficult to retrieve due to their rarity in anatomic pathology archives. Since variation of methylation level also varies within single species, further studies are needed to confirm our preliminary data on cows, horses, badgers, porcupines, and eventually, other mammals, to enrich more comprehensive and powerful results. Most of our data are based on SCCs coming from oral tissue specimens of humans, cats, and dogs, so only a partial epigenetic landscape of UCNE in mammals has been pointed out in our study. A few of the SCCs here investigated had different sites of origin (skin, vulva, mandible, or lung), however recent findings reported that SCCs from various body sites have common epigenetic and genetic determinants, pointing to a unified perspective of the disease and potential new avenues for prevention and treatment [35]. Moreover, the role of DNA methylation in UCNE should be further compared to the expression levels, but this will be feasible using only fresh/frozen tissues which are difficult to retrieve, especially for those species that rarely are admitted to veterinary clinics.

## 5. Conclusions

Our findings indicate that UCNEs are hypermethylated in human SCCs, and this behavior is also conserved among different species of mammals. Further comparative studies are needed to investigate the molecular similarities between human and animal SCCs and their potential usefulness to understand and combat this neoplasm.

## Figures and Tables

**Table 1:** Clinical details of mammalian species other than human included in this study.

ID	Sample	Diagnosis	Species	Breed	Location	Sex	Age
AP15289	FFPE	SCC	*Felis catus* (Cat)	Domestic short-haired	Skin (head)	Fs	9 y
AP8012	FFPE	SCC	*Felis catus* (Cat)	Domestic short-haired	Skin (chin)	Mc	10 y
AP11378	FFPE	SCC	*Felis catus* (Cat)	Domestic short-haired	Skin (ear)	Fs	12 y
AP11306	FFPE	SCC	*Felis catus* (Cat)	Domestic short-haired	Oral cavity	Fs	12 y
AP18329	FFPE	SCC	*Felis catus* (Cat)	Domestic short-haired	Oral cavity	Mc	11 y
AP18369	FFPE	SCC	*Felis catus* (Cat)	Domestic short-haired	Oral cavity	Fs	13 y
AP4925	FFPE	SCC	*Felis catus* (Cat)	Domestic short-haired	Oral cavity	Fs	19 y
AP9641	FFPE	SCC	*Felis catus* (Cat)	Domestic short-haired	Oral cavity	Mc	16 y
AP9960	FFPE	SCC	*Felis catus* (Cat)	Persian	Oral cavity	Mc	11 y
AP17845	FFPE	SCC	*Felis catus* (Cat)	Domestic short-haired	Oral cavity	Mc	11 y
AP17919	FFPE	SCC	*Felis catus* (Cat)	Domestic short-haired	Oral cavity	Fs	10 y
AP13806	FFPE	SCC	*Felis catus* (Cat)	Domestic short-haired	Oral cavity	Mc	15 y
AP17595	FFPE	SCC	*Felis catus* (Cat)	Thai	Oral cavity	Fs	12 y
AP17714	FFPE	SCC	*Felis catus* (Cat)	Domestic short-haired	Oral cavity	Mc	9 y
AP18102	FFPE	SCC	*Felis catus* (Cat)	Domestic short-haired	Oral cavity	Mc	12 y
AP768	FFPE	SCC	*Felis catus* (Cat)	Domestic short-haired	Oral cavity	Fs	15 y
AP8694	FFPE	SCC	*Felis catus* (Cat)	Domestic short-haired	Oral cavity	Fs	12 y
AP16359	FFPE	SCC	*Canis lupus familiaris* (Dog)	Beagle	Prepuce	M	12 y
AP17735	FFPE	SCC	*Canis lupus familiaris* (Dog)	Labrador retriever	Skin	F	11 y
AP16030	FFPE	SCC	*Canis lupus familiaris* (Dog)	Labrador retriever	Oral cavity	Fs	14 y
AP7483	FFPE	SCC	*Equus ferus caballus* (Horse)	Paint horse	Vulva	F	15 y
AP12812	FFPE	SCC	*Bos taurus* (Bovine)	Holstein fresian	Eyelid	Mc	8 y
S17_3828	FFPE	SCC	*Meles meles* (European badger)		Lung	F	14 y
S18_5874	FFPE	SCC	*Hystrix cristata* (Porcupine)		Mandible	F	14 y
AP10728	FFPE	Stomatitis	*Felis catus* (Cat)	Domestic short-haired	Oral cavity	Mc	9 y
AP2633	FFPE	Stomatitis	*Felis catus* (Cat)	Domestic short-haired	Oral cavity	Fs	10 y
AP5978	FFPE	Stomatitis	*Felis catus* (Cat)	Domestic short-haired	Oral cavity	Fs	7 y
AP847	FFPE	Stomatitis	*Felis catus* (Cat)	Domestic short-haired	Oral cavity	Fs	12 y
AP18349	FFPE	Stomatitis	*Felis catus* (Cat)	Domestic short-haired	Oral cavity	Mc	8 y
AP16102	FFPE	Stomatitis	*Felis catus* (Cat)	Domestic short-haired	Oral cavity	Mc	17 y
AP17827	FFPE	Stomatitis	*Felis catus* (Cat)	Domestic short-haired	Oral cavity	Mc	9 y
AP17936	FFPE	Stomatitis	*Felis catus* (Cat)	Domestic short-haired	Oral cavity	Fs	4 y
AP18136	FFPE	Stomatitis	*Felis catus* (Cat)	Domestic short-haired	Oral cavity	Mc	5 y
AP18404	FFPE	Stomatitis	*Felis catus* (Cat)	Domestic short-haired	Oral cavity	Fs	8 y
AP2115	FFPE	Stomatitis	*Felis catus* (Cat)	Domestic short-haired	Oral cavity	Fs	12 y
AP498	FFPE	Stomatitis	*Felis catus* (Cat)	Domestic short-haired	Oral cavity	Mc	1 y
AP7692	FFPE	Stomatitis	*Felis catus* (Cat)	Domestic short-haired	Oral cavity	Fs	13 y
AP17076	FFPE	Stomatitis	*Felis catus* (Cat)	Domestic short-haired	Oral cavity	Mc	10 y
CAT-1	Brushing	Norm	*Felis catus* (Cat)	Domestic short-haired	Oral cavity	Mc	15 y
CAT-2	Brushing	Norm	*Felis catus* (Cat)	Domestic short-haired	Oral cavity	Fs	10 y
CAT-3	Brushing	Norm	*Felis catus* (Cat)	Domestic short-haired	Oral cavity	Fs	9 y
CAT-4	Brushing	Norm	*Felis catus* (Cat)	Domestic short-haired	Oral cavity	Mc	10 y
CAT-5	Brushing	Norm	*Felis catus* (Cat)	Domestic short-haired	Oral cavity	Fs	9 y
CAT-6	Brushing	Norm	*Felis catus* (Cat)	Persian	Oral cavity	Fs	11 y
CAT-7	Brushing	Norm	*Felis catus* (Cat)	Domestic short-haired	Oral cavity	Mc	12 y
DOG-1	Brushing	Norm	*Canis lupus familiaris* (Dog)	Border Collie	Oral cavity	Mc	5 y
DOG-2	Brushing	Norm	*Canis lupus familiaris* (Dog)	Golden Retriever	Oral cavity	Mc	10 y
DOG-3	Brushing	Norm	*Canis lupus familiaris* (Dog)	Newfoundland	Oral cavity	Fs	9 y
DOG-4	Brushing	Norm	*Canis lupus familiaris* (Dog)	Golden Retriever	Oral cavity	F	7 y
DOG-5	Brushing	Norm	*Canis lupus familiaris* (Dog)	Golden Retriever	Oral cavity	F	5 y
HORSE-1	Brushing	Norm	*Equus ferus caballus* (Horse)	Italian Saddle	Oral cavity	Mc	16 y
HORSE-2	Brushing	Norm	*Equus ferus caballus* (Horse)	Italian Saddle	Oral cavity	Mc	9 y
HORSE-3	Brushing	Norm	*Equus ferus caballus* (Horse)	Italian Saddle	Oral cavity	Mc	8 y
HORSE-4	Brushing	Norm	*Equus ferus caballus* (Horse)	Italian Saddle	Oral cavity	F	10 y
HORSE-5	Brushing	Norm	*Equus ferus caballus* (Horse)	Italian Saddle	Oral cavity	F	9 y
BOV-1	Brushing	Norm	*Bos taurus* (Bovine)	Holstein fresian	Oral cavity	F	2 y
BOV-2	Brushing	Norm	*Bos taurus* (Bovine)	Holstein fresian	Oral cavity	F	1 y 6 m
BADGER-1	Brushing	Norm	*Meles meles* (European badger)		Oral cavity	F	Unknown (adult)

**Table 2:** Genomic regions investigated in this study (reference genome: hg38; gene annotations were retrieved from UCNE database [2] and from Bajerano database [1]).

UCNE Bejerano	UCNE ID	UCNE Name	Upstream Gene	Within Gene	Downstream Gene	Primer For	Primer Rev	Genome Coordinates (hg38)	Number of CpG	Amplicon Length
uc.160+	27423	OTP_Lukas	*AK128395*	*NA*	*AP3B1*	TTTTTGATTTTAGGTGGAATTAGGAG	TACATTAAAACCTCAATTAATTAACCCTTA	Chr5+: 77972981−77973184	6	204
uc.283+	6661	DRGX_Toru	*DRGX*	*NA*	*ERCC6*	TTGGGTGAAAATTAAATTTTAAAGTAT	AAAAAACACTTAATAATACAAAAAACC	Chr10+: 49396679−49396856	6	178
uc.283 Prom	6661	DRGX_Toru	*DRGX*	*NA*	*ERCC6*	TGTTTTTAGTAAGGGGTTTTTGAATT	AAAATTTAATTTTCACCCAAACAAA	Chr10+: 49396546−49396698	6	153
uc.416+	NA	NA	*HOXB4*	*HOXB5*	*HOXB6*	GTTTTAAAGTGGTTGGAGGAGGT	CTATCAATTACTAAATTATAACAATAACAA	Chr17+: 48593480−48593629	7	150
uc.339+	NA	NA	*ATP5G2*	*NA*	*KIAA1536*	GTTATTATAGGAATGTAATTTTGTT	CCTTTCAAAAACTCAAAACCATAAA	Chr12+: 53677385−53677558	9	174
uc.270+	35244	NA	*AB040954*	*MAPKAP1*	*LOC51145*	GTGATGGGTATTTAGAGAGTTGGTTAT	CTTACCAAAAATCATTATTCACCCT	Chr9+: 125541877−125541998	7	122
uc.299+	NA	NA	*HIF1AN*	*PAX2*	*C10orf6*	TTTTGTTGTGTGTGGGGTGT	CCTCACCTACCCAAAATTTTACTAAC	Chr10+: 100749700−100749920	12	221
uc.328+	NA	NA	*ELP4*	*PAX6*	*RCN1*	TTTGATATTTGTTTTTAAAATAAAATTAGT	ACAAAATAACAAAACTACCACAAAC	Chr11+: 31804130−31804274	6	145

**Table 3:** Best and worst AUC values for all the seven UCNEs; the number indicates the position on the genome taking into account hg38 as a reference.

UCNE	Best Position	Best AUC Value	Worst Position	Worst AUC Value	Best to Worst AUC Gap
uc.160	77268955	0.783	77268898	0.725	0.058
uc.270	128304242	0.814	128304236	0.589	0.225
uc.283	50604683	0.824	50604779	0.653	0.171
uc.299	102509546	0.752	102509564	0.686	0.066
uc.328	31825751	0.713	31825744	0.624	0.089
uc.339	54071206	0.826	54071264	0.742	0.084
uc.416	46670879	0.843	46670918	0.679	0.164

## Supplementary Materials

The following are available online at https://www.mdpi.com/2073-4409/9/9/2092/s1: Figure S1: Methylation plot (left) and boxplot (right) comparing canine SCC and normal oral epithelium in healthy dogs; Figure S2: Methylation plot (left) and boxplot (right) comparing equine SCC and normal oral epithelium in healthy horses; Figure S3: Comparison among SCCs from different species: methylation plot (left) and boxplot (right); Figure S4: Comparison among normal samples from different species: methylation plot (left) and boxplot (right); Table S1: Summary Table with mean, standard deviation, minimum, maximum, and *p* value of the Kruskal–Wallis test for each position and group of samples comparing all SCCs from all the species together vs. normal samples and feline stomatitis; Table S2: Summary Table comparing human normal donors and SCCs; Table S3: Summary Table with mean, standard deviation, minimum, maximum, and *p* value of the Kruskal–Wallis test for feline SCC vs. feline normal samples and feline stomatitis.
