# Supplementary figures and images for: An Evolutionary Cancer Epigenetic Approach Revealed DNA Hypermethylation of Ultra-Conserved Non-Coding Elements in Squamous Cell Carcinoma of Different Mammalian Species
Source: Cells. 2020 Sep 13;9(9):2092. doi: 10.3390/cells9092092 (PMC7565279; doi:10.3390/cells9092092)

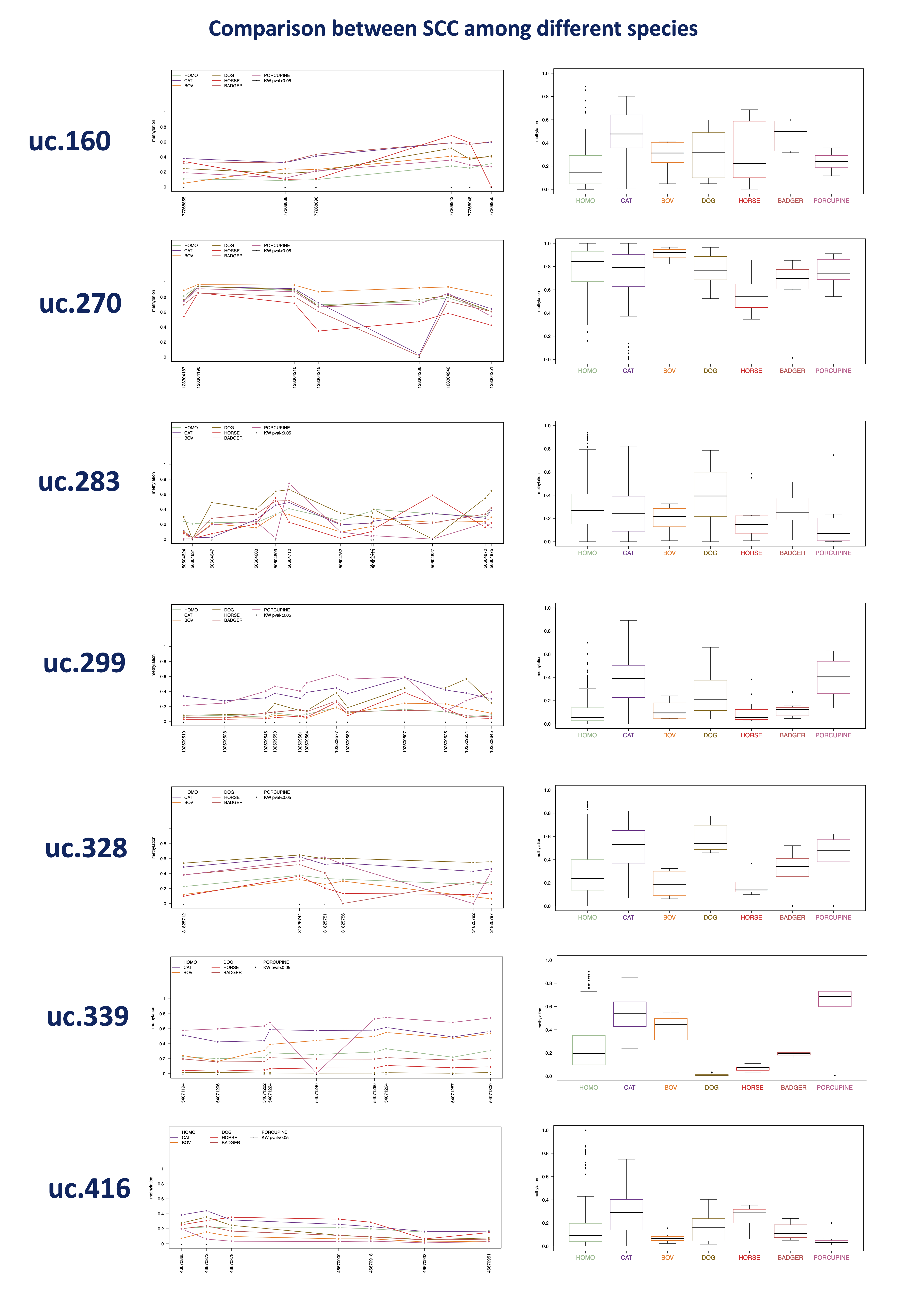

Supplement: Supplementary file 1 [file cells-09-02092-s001.zip › SuppFiles/Fig.S4.jpg]

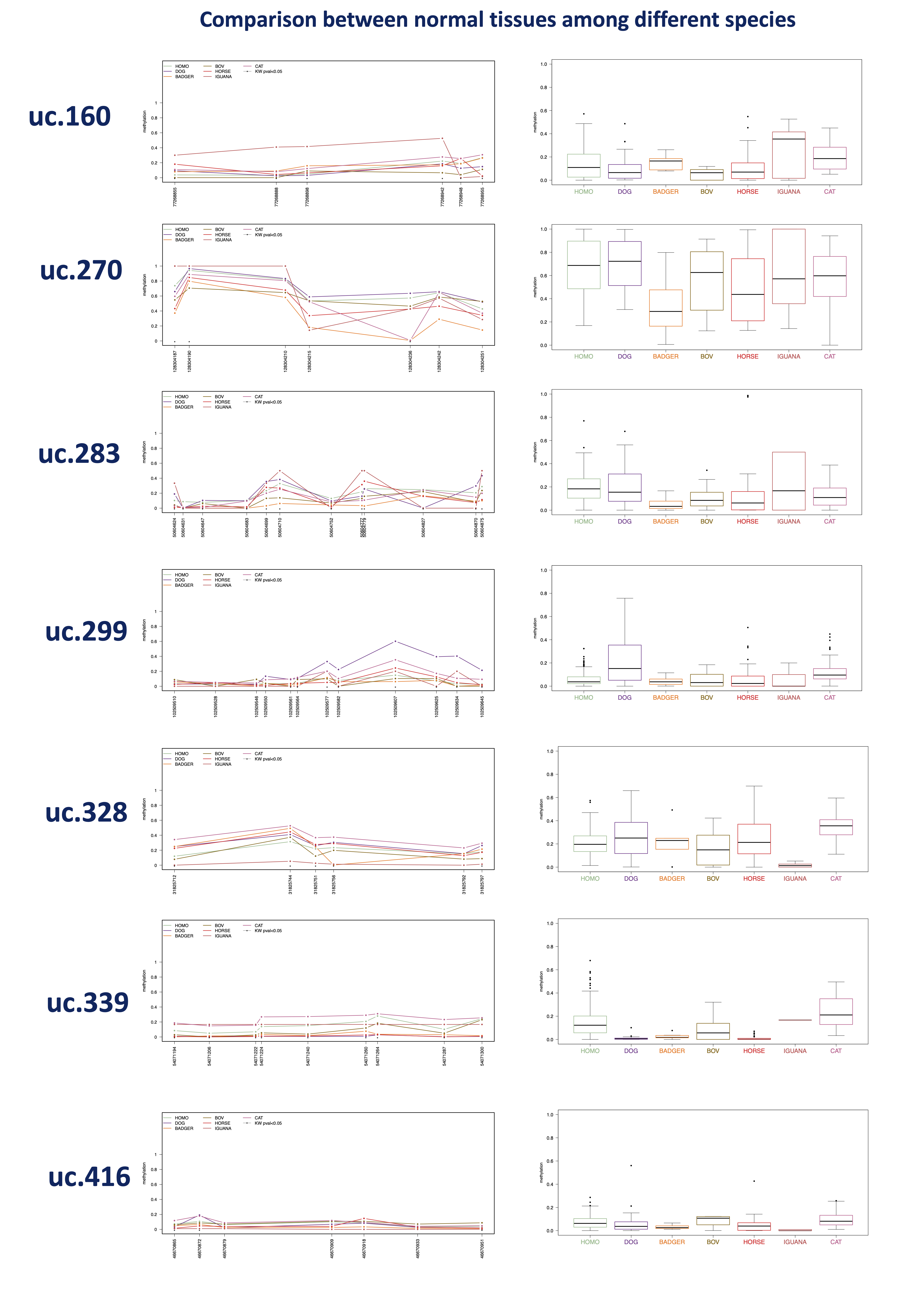

Supplement: Supplementary file 1 [file cells-09-02092-s001.zip › SuppFiles/Fig.S5.jpg]

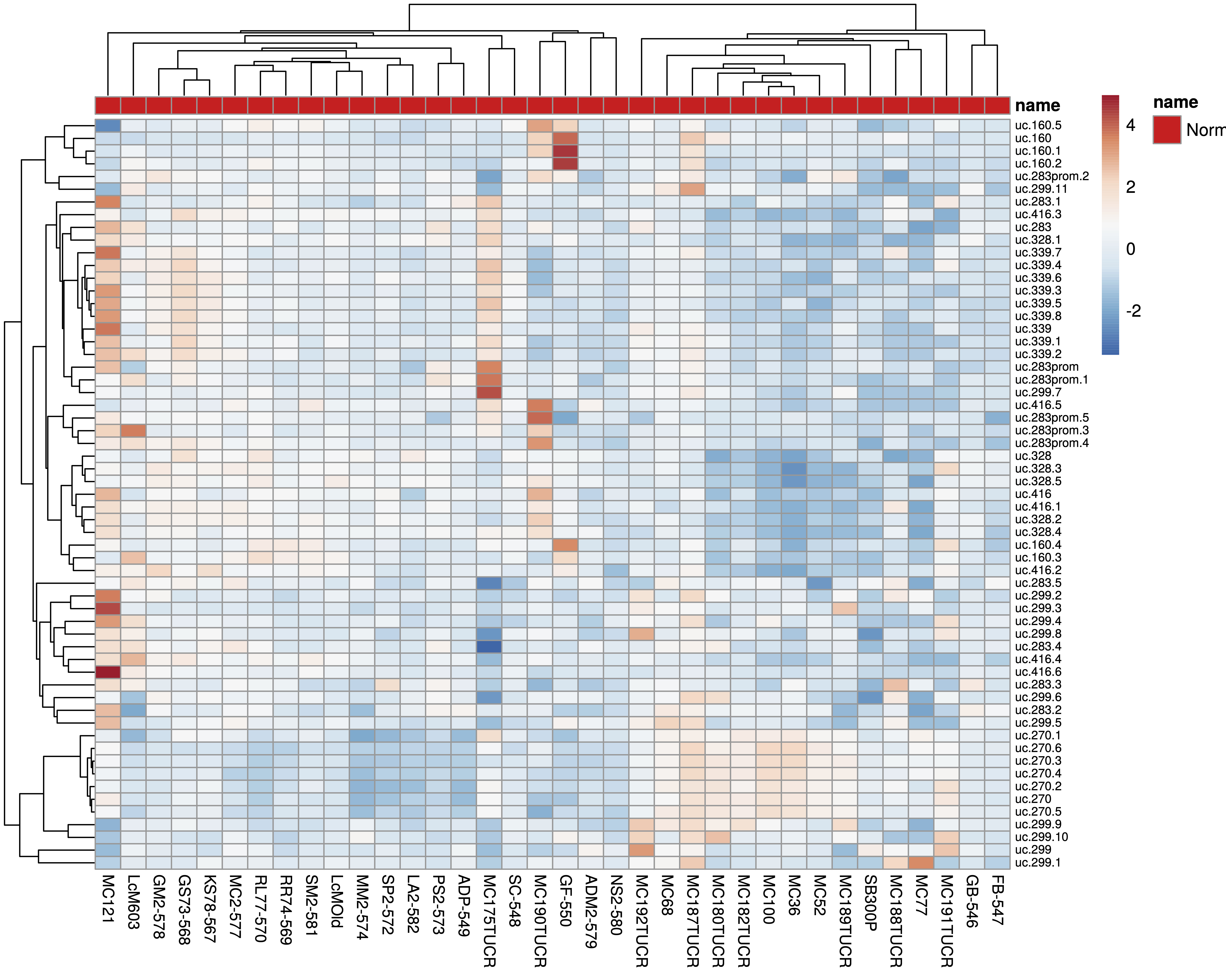

Supplement: Supplementary file 1 [file cells-09-02092-s001.zip › SuppFiles/Fig.S1.jpg]

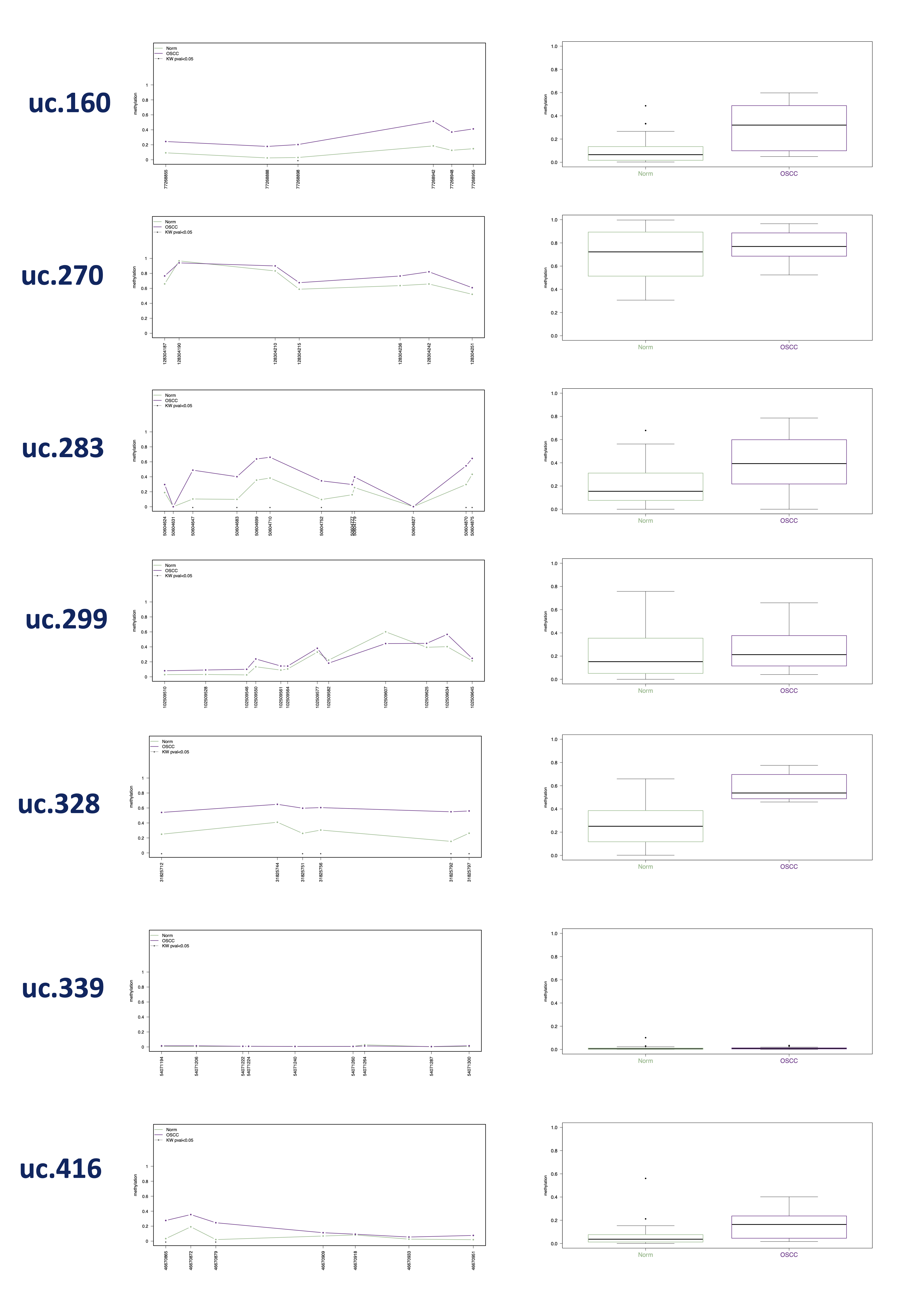

Supplement: Supplementary file 1 [file cells-09-02092-s001.zip › SuppFiles/Fig.S2.jpg]

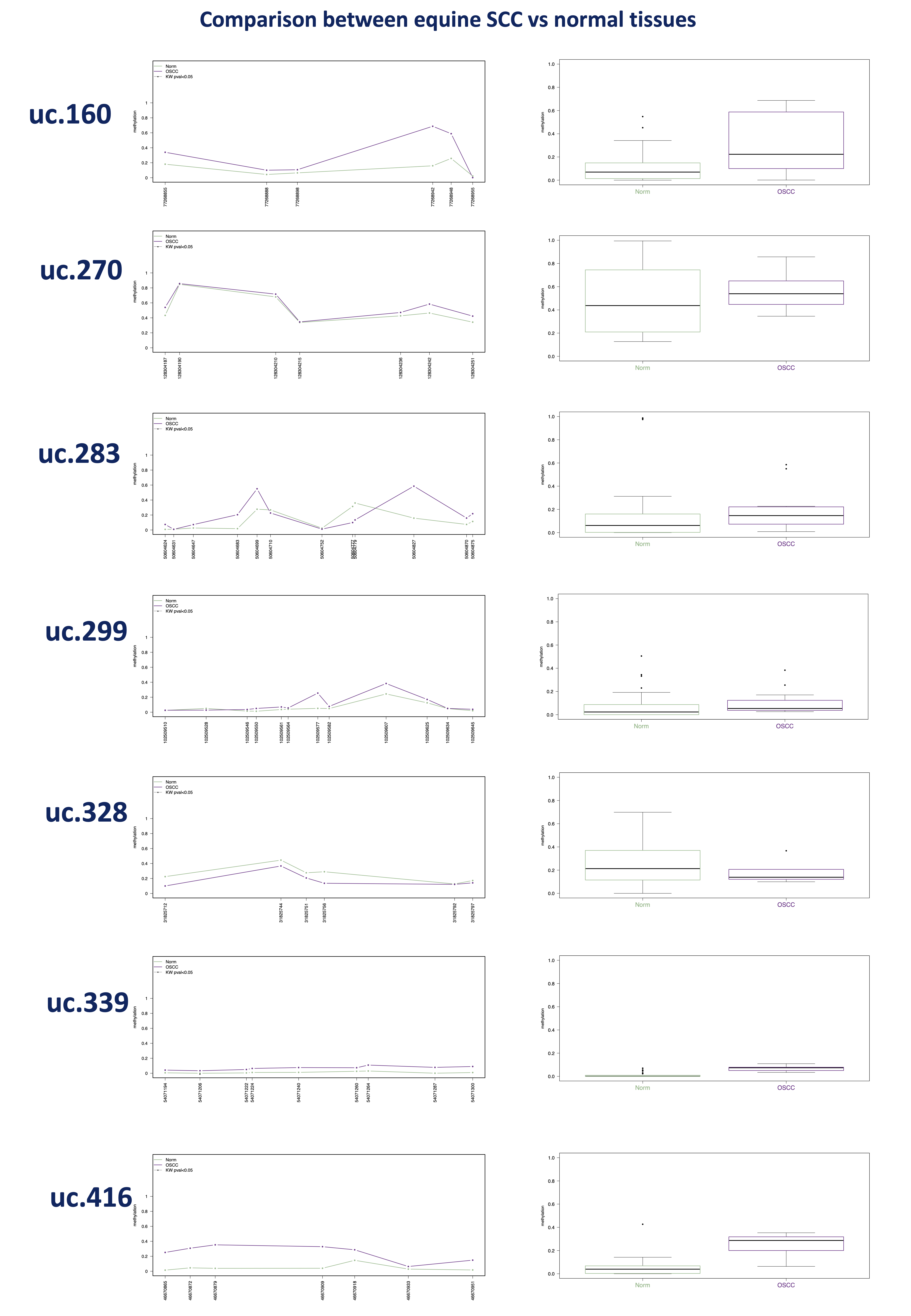

Supplement: Supplementary file 1 [file cells-09-02092-s001.zip › SuppFiles/Fig.S3.jpg]
